# Credibility revolution: pursuing a balanced and sustainable approach, without dogmas, without magic elixirs

**DOI:** 10.3389/fpsyg.2025.1581160

**Published:** 2025-04-16

**Authors:** Antonino Vallesi

**Affiliations:** ^1^Department of Neuroscience, Università degli Studi di Padova, Padova, Italy; ^2^Padova Neuroscience Center, Università degli Studi di Padova, Padova, Italy

**Keywords:** replicability crisis, credibility revolution, scientific discovery, creativity, psychological theories, open science

## 1 Introduction: replicability crisis

In this Editorial Grand Challenge, I will first summarize some of the multiple (intrinsic and extrinsic) reasons why cognitive psychology, along with many other scientific fields, has experienced a replicability crisis. I will then illustrate some of the proposed remedies that are leading to a credibility revolution. I will highlight that the proposed best practices for complex epistemological problems, while necessary, also need to be critically evaluated to ward off potentially new concerns. Finally, I will advocate for a new balance to ensure methodologically sound and credible science while also safeguarding scientific sustainability, creativity and enthusiasm.

In the history of psychology, periods of vibrant discovery and exciting research have occasionally been perturbed by troubling waves of replicability crisis, the most recent one of which is still ongoing (Lakens, 2023; Open Science Collaboration, 2015). While cognitive psychology appears to be slightly less affected by the replicability crisis than social psychology (Open Science Collaboration, 2015), it has certainly not been immune to similar concerns.

First, publications with small sample sizes and inadequate statistical power often led to findings that could not always be reliably reproduced (Button et al., 2013). Second, the pressure to publish novel and positive results, coupled with distorting incentives, has fostered *questionable* research practices (or even fraud, in the worst cases), such as for instance selective reporting and p-hacking, in which some researchers may, more or less deliberately, manipulate or select data to achieve statistically significant outcomes (e.g., Lilienfeld, 2017; Nosek et al., 2012). Additionally, complex experimental designs, researchers' degrees of freedom and variability in methodological practices have contributed to inconsistencies across studies. The prevalence of a confirmation bias, that is, the tendency of researchers to favor data that support their hypotheses, has further exacerbated this issue. Moreover, a lack of transparency in data sharing and reporting of methodological details has hindered efforts to accurately replicate findings.

These challenges are currently prompting a massive cultural paradigm shift that would imply entirely rethinking the research process. It is not surprising that a growing movement, largely and meritoriously driven by the new generation of researchers, is advocating for more rigorous and transparent scientific practices. This shift is a virtuous reaction to the replicability crisis (e.g., Lindsay, 2020). These practices include for instance, (i) registering reports or at least pre-registering studies to minimize questionable practices such as Harking (Kerr, 1998), cherry-picking and confirmation bias (e.g., Clark et al., 2022a), (ii) implementing open data initiatives to increase transparency and maximizing collective scientific efforts (e.g., Mayernik, 2017; Rasti et al., 2025), and (iii) conducting replication efforts through coordinated multi-laboratory projects to achieve the large sample sizes necessary for sufficient statistical power. Therefore, awareness of the replicability crisis is currently driving positive and praiseworthy changes in the methods, procedures and, importantly, policies that will eventually and ideally benefit the credibility of the scientific community as a whole (e.g., Korbmacher et al., 2023). However, as I will argue throughout this contribution, one would be naïve to think that the proposed solutions to these complex epistemological problems are always straightforward and without their own drawbacks. Any new remedy needs to be critically evaluated and a new balance sought.

## 2 Multiple contributions to the replicability crisis

As a first consideration, it is undeniable that the replicability crisis can be, at least partially, attributed to a series of questionable research practices that have been commonly used, tolerated, or even favored by standard practices in the field and by ill-conceived academic incentives. However, we should not overlook other potential factors that may also have contributed to the replicability crisis in our specific field. Indeed, replicability issues may also stem, in part, from deeper epistemological and methodological problems.

The social sciences, and cognitive psychology is no exception, are concerned with objects of investigation that cannot be observed directly but are captured primarily through latent variables and constructs. These constructs require careful, and often challenging, operationalization in order to be scientifically attacked through some measurable proxies. Because cognitive processes are inherently “fuzzier” and more ambiguous compared to more basic biological processes, there is typically no single best operationalization of a given construct to test, and ontological definitions require additional effort. To take an example from cognitive psychology used by Poldrack and Yarkoni (2016), performance on N-back tasks and OSPAN tasks shows weak, and often non-significant, correlations (Kane et al., 2007; Oberauer, 2005; Roberts and Gibson, 2002). This is unsettling as both tasks are designed to assess the same cognitive construct, that is, working memory. Such discrepancies suggest issues such as ambiguous operational definitions and task impurity (Kane et al., 2007), which contribute to the entropy in the literature. Some promising remedies have been proposed. These include the Cognitive Atlas Project (Poldrack and Yarkoni, 2016), a collaborative project to develop a knowledge base that represents the current state of cognitive science, or the Cognitive Paradigm Ontology (Turner and Laird, 2012), a systematic collection of cognitive paradigms for functional neuroimaging applications. While these tools are commendable, there is a risk that only global inferences can be made. This would happen if the tasks included in these databases were too general and lacked subtask-specific manipulations. Indeed, despite their methodological idiosyncrasies, subtle experimental manipulations are often more theoretically meaningful than the common features across tasks.

Additionally, the findings obtained need to be interpreted, with more than one account often being equally plausible at the same time, thus lowering internal validity. This produces uncertainty about which interpretation of the findings might be the most accurate, increasing the likelihood that biased conclusions will be preferred instead. A related consequent issue is the so-called *motivated* research, which occurs when factors unrelated to accuracy influence how scientists engage with existing data, generate new hypotheses, collect, analyze and interpret data, and communicate research outcomes to their peers and the general audience (e.g., Clark et al., 2022b; Kunda, 1990). These factors include confirmation biases and the need for social and moral approval of specific research topics and conclusions.

Furthermore, delving deeper into the core of the issue, the inherent variability of the object of investigation is another major challenge in the social sciences, which also include cognitive psychology. The manifestation of cognitive processes can indeed vary considerably, not only between individuals but also within individuals (e.g., Dhawale et al., 2017; Fiske and Rice, 1955; Kahana et al., 2018; MacPherson et al., 2019), with variability being a key aspect of cognition that cannot be dismissed (or at least not exclusively) as a *measurement error* (Judd et al., 2024). Indeed, variability is not only spuriously caused by ill-defined operationalizations of cognitive constructs, imprecise measurements of their proxies (e.g., behavior), violations of statistical assumptions, power issues, and more generally, poor experimental control and other methodological flaws (e.g., Clark and Watson, 2019; Podsakoff et al., 2012). There are also other fundamental sources of variability linked to the very nature of mental processes.

Natural fluctuations characterize the manifestations of mental functions such as perception, emotion, memory, executive functions, and decision-making, to name a few. This variability arises from numerous factors, which include psychobiological variables, genetic differences, developmental stages, but also situational contexts and environmental influences (e.g., epigenetics).

Specifically, cognitive performance can be affected by an individual's mood (Chepenik et al., 2007), motivation (Braver et al., 2014), stress (Steinhauser et al., 2007), sleep deprivation (Killgore, 2010), and prior experience (Pastukhov and Braun, 2011). Moreover, the brain's complex neural architecture and the dynamic nature of its networks contribute to this variability, as its connectome is constantly shaped and reshaped by new experiences, providing the neural foundation for an ever-changing cognitive life (e.g., Dosenbach et al., 2010; Kolb and Gibb, 2011; Seung, 2012; Tost et al., 2015). Acknowledging, and ideally accounting for, this intrinsic variability and lability of the object of investigation in cognitive psychology (i.e., mental processes) is essential for developing robust, or at least plausible, theories and applications in cognitive psychology and neuroscience.

Additionally, many findings cannot be easily generalized across different contexts (e.g., cultural, spatial, temporal) or experimental conditions (Stroebe and Strack, 2014). Indeed, the environmental or experimental context can affect both how and to what extent a cognitive process is activated and expressed. An example is provided in the work by Hommel et al. (2012). Their study showed that cognitive control can be, somewhat counterintuitively, more effective when exercised in the context of the acoustic noise typical of fMRI sequences than in standard settings. This specific example raises more general considerations about the potential applicability of Heisenberg's uncertainty principle in cognitive neuroscience, where the object of investigation (the mind) is being transformed by the very tools used to study it. A more classic example of this issue is the *Hawthorne* effect, in which individuals alter their behavior because they are aware of being observed and measured (e.g., Adair, 1984). Although, ironically enough, this famous phenomenon is probably not fully reliable, or at least not in its initial reports (Letrud and Hernes, 2019; Levitt and List, 2011), it represents just one example of the broader (but often neglected) modulatory role of participants' motivation and *demand characteristics* in task performance (e.g., Orne, 1962; Weber and Cook, 1972).

To summarize this section, while variability can be seen as an obstacle to scientific progress, as it can lead to average null effects, uncovering its intrinsic (but also exogenous) sources can also have a leveraging effect on theory development. Indeed, variability could be exploited to better understand inter-individual differences in cognition and to advance cognitive theories (e.g., Langerock et al., 2025; Miller and Schwarz, 2017; Wang et al., 2012; cf., Rowe and Healy, 2014).

## 3 Remedies for the replicability crisis and new concerns of the credibility revolution

One potential concern with the proposed remedies for the replicability crisis is that, while highly desirable in principle, some of them conceal other risks that should at least be acknowledged, if not actively countered. As the Latin writer Horace once wrote: “Est modus in rebus”, which reminds us to pursue balance in life by avoiding deleterious extremes. As will become clear from the following, the coveted solutions proposed to overcome the replicability crisis are also subject to this caveat. Therefore, we need to avoid applying them uncritically.

### 3.1 Data-driven approaches in cognitive psychology: potential risks and antidotes

While the widespread availability of large open datasets promotes reproducibility and collective efficiency, it also inevitably encourages data mining and exploratory approaches. Far be it from me to want to unleash or incite a *witch hunt*, but such a data-driven mindset (which could bring to a so-called *fishing expedition* attitude) can sometimes temptingly lead to the *post hoc* generation of hypotheses (HARKing) and, even worse, to a lack of theoretical depth. A data-driven attitude is also encouraged by the unprecedented, exponential expansion of Machine Learning and other Artificial Intelligence (AI) algorithms. AI can undoubtedly enhance the efficient exploration and analysis of complex scientific data in a number of ways, such as identifying patterns and trends, allowing predictive modeling, integrating multidimensional data and so on, accelerating data-driven discoveries including in the field of cognitive psychology. However, would powerful but opaque AI algorithms always help enhance our theoretical understanding of underlying cognitive mechanisms? And, in case this is achievable, could this AI-based understanding then generalize to new datasets? Although the debate in the field is currently heated with these and other open questions, it would definitely benefit from a more extensive and deeper epistemological reflection, hopefully with contributions also coming from cognitive psychology.

One proposed solution to the risks of a lenient data-driven attitude is the pre-registration of studies, which forces researchers to truly specify their *a priori* hypotheses in advance (and to clearly label as *exploratory* all the other hypotheses that are generated *a posteriori* with respect to the pre-registration). However, these and other credibility-enhancing practices are still adopted on a voluntary basis and are not yet routinely applied as gold standards. Additionally, pre-registration alone (as a mere bureaucratic formality) is not sufficient. It would be equally important to address the deeper issue of the theoretical crisis: the hypotheses that are pre-registered could still be purely empirical in nature or, even worse, poorly formulated, unjustified and without strong theoretical foundations. Thus, pre-registering hypotheses helps to reassure the scientific community that our thinking remains unbiased but it does not in itself substitute theoretical and logic-based reasoning. Simply pre-registering a hypothesis, as a sort of *fig leaf*, without substantiating it with a solid theoretical rationale serves little purpose and does not in itself enhance the credibility of the hypothesis (Oberauer and Lewandowsky, 2019). Moreover, it would be fine to candidly admit that a given study was exploratory, especially if at the beginning of a new line of research, as long as the data obtained could inspire theory development, new hypotheses, and further research to test those hypotheses and refine the theory.

An excessive focus on trying to empirically reproduce or replicate published effects or phenomena, while extremely valuable to reshape the trust and the foundation of what we already know about human cognition, if an end in itself, can divert attention away from developing robust theories (Oberauer and Lewandowsky, 2019). Proposing strong theories would allow scholars to formulate credible and specific hypotheses, which would benefit scientific progress (Clark et al., 2022b). Developing accurate and comprehensive theories or at least models of cognitive processes is inherently challenging due to their fuzzy nature and complexity. Thus, theories and models need to consider a broad spectrum of variables and interactions, which necessitates advanced computational approaches, biological plausibility and extensive experimental testing (e.g., Busemeyer and Diederich, 2010; Turner et al., 2017). This process could benefit from collective, multidisciplinary scientific coordination toward common, challenging goals (Brignol et al., 2024; Rasti et al., 2025).

One special type of scientific coordination that has been proposed as a remedy for the lack of a theory-oriented mindset is represented by adversarial collaborations (sometimes referred to as “coopetition”; Clark et al., 2022a; Ellemers et al., 2020). Although challenging, engaging with those who propose alternative theories can be scientifically rewarding in the long run (see Cowan et al., 2020; Mellers et al., 2001, for some examples). These collaborations require clear, testable hypotheses, mutual understanding of differing viewpoints, and methods that can distinguish and potentially falsify both hypotheses. This process limits researchers' biases and beliefs, enhances the integrity of methodological approaches, and effectively advances debates. By committing to methods before testing, even better when coupled with best practices such as pre-registering, open data and replication, scholars reduce the risk of *post-hoc* criticism or hiding unwanted results. This would also make the findings more credible to the scientific community. More plausible hypotheses will prevail, and incorrect ones will be falsified sooner, preventing wasted time and resources and increasing the reliability and credibility of scientific findings.

Relying on increasingly sophisticated methods to analyze rich and complex data poses similar risks. The field of neuroimaging can be used as an example. While advances in neuroimaging data analysis have provided valuable insights into brain activity, understanding how these findings are related to cognitive processes is still difficult. Explaining how complex mental functions arise from brain regions and neural networks has traditionally been a major challenge (e.g., Coltheart, 2006; Hommel and Colzato, 2017; Niv, 2021). More specifically, while recent advances in the field of connectomics in cognitive neuroscience (e.g., graph theory) have significantly enhanced our ability to explain the complexity of brain organization, at least in principle, clear relationships between topological indices of network integration and segregation and cognitive processes have yet to be fully established (Litwińczuk et al., 2023). These relationships would be crucial for assessing the biological plausibility and behavioral relevance of these highly derived, network-based measures.

Complicating matters, it is acknowledged in the neuroimaging literature that behavioral data are not always characterized with sufficient fidelity to support robust and reliable brain-behavior correlations (Tiego and Fornito, 2022). Additionally, some common interpretations of cognitive neuroscience findings are unjustified because they rely on flawed reasoning or other unproven assumptions. A well-known example of flawed reasoning is reverse inference, that is, deducing the involvement of particular mental processes solely based on observed patterns of brain activation, without the constraints of elegant experimental manipulations that would enhance internal validity (Kriegeskorte et al., 2009; Poldrack, 2006). As another example, many compensatory cognitive interpretations of brain patterns in older adults are not fully justified unless data are analyzed longitudinally (e.g., Pudas et al., 2013).

Another potential drawback of widely available open data is the inherent risk of *salami slicing*, in which data from a research study that should form a single publication are divided into several smaller publishable units. Salami slicing, which has long been considered a questionable and unethical research practice in various fields (Karlsson and Beaufils, 2013; Siegel and Baveye, 2010; Spielmans et al., 2010) remains a significant concern, and even more so in the era of open science. This practice can lead to various issues, such as consuming excessive resources during the editorial and review process and overwhelming science readers. It can also lead to an inflated number of articles cluttering the literature, increasing the risk of publication bias, and distorting effect sizes in meta-analyses. To overcome this issue, the same or overlapping data samples should always be properly acknowledged as such in different publications (e.g., Hilgard et al., 2019; Urbanowicz and Reinke, 2018).

Furthermore, it is also worth mentioning that the risk of wasting resources and producing flawed science is amplified if the tasks used to collect behavioral data in large open datasets were not carefully designed and suffer from some error.

### 3.2 Potential side effects of the credibility revolution and excessive methodological control

A well-known trade-off exists between experimental control and generalizability. On the one hand, more attention to well-designed experimental paradigms that account for confounding factors when trying to fully characterize a psychological effect is desirable to increase internal validity (e.g., Verbruggen et al., 2019; Viviani et al., 2024), and hopefully also to enhance scientific credibility and replicability. On the other hand, excessive methodological control in research poses a significant risk, as it can lead to a decrease in generalizability, that is, external and ecological validity (Shadish et al., 2002). In other words, the more tightly controlled experiments become, the less accurately their findings reflect real-world conditions, making it difficult to apply the results to broader contexts or diverse populations. This undermines the relevance and applicability of the research, limiting its potential impact outside the highly controlled environment in which it was conducted.

Another potential risk of placing too much emphasis on the remedies for the replicability crisis is a reduction in the enthusiasm for exploration, creativity and discovery, which are also essential aspects of the scientific process that complement methodological rigor. Focusing too much on strict standards, while being undeniably vital for overcoming the pressing credibility crisis, may also lead to excessive caution, hindering divergent thinking and the serendipitous attitude that drives new discoveries (Kaufman and Glǎveanu, 2018; Ness, 2015). In principle, the majority of researchers would support recommendations, best practices, and, more generally, to virtuous organized skepticism, in the hope that the scientific community would universally adopt them (Anderson et al., 2007). However, when these begin to become imposed *norms*, a looming risk is that they may lead to additional paperwork in fulfilling the already hypertrophic and self-referential accountability and evaluation processes. The latter require additional time and mental energy, limited resources that researchers would prefer to devote, with much more motivation and enthusiasm, to their actual research activities. If new practices become excessively demanding due to an increased bureaucratic burden, they risk reducing the time and resources available for creative activities (Kaufman and Glǎveanu, 2018). In other words, with excessive constraints, creativity could also be suppressed (Medeiros et al., 2014). This risk within the scientific field should be considered in addition to the already observed decline in creativity in the general population (Kim, 2011).

In other words, while best practices in science are indispensable and non-negotiable, the burdensome bureaucracy and other possible downsides that might accompany their implementation should be kept strictly in check, and new incentives should be envisioned that could promote slower but more accurate science and allow for more quality time. Scientists are therefore compelled to closely monitor the tendency toward hyper-bureaucratization of the scientific process and to continually seek a new balance between the apparently conflicting, yet equally essential needs of methodological rigor and scientific creativity (Ness, 2015; Scheffer, 2014; Simonton, 2004), while moving away from purely quantity-based institutional evaluation processes and incentives.

## 4 Concluding remarks

The awareness of a massive replicability crisis has been a slap in the face for the scientific community. It is undeniable that we can no longer continue with business as usual and that remedies to address this crisis must be implemented. However, as I have tried to argue here, this cannot be done naively or uncritically, as any new practice could also bring its own drawbacks, including changes in how we value scientific research. It has been shown, for instance, that awareness of replication failures and criticism of questionable research practices can diminish trust in past research (Anvari and Lakens, 2018). Worse, being informed about potential solutions, such as increased transparency, may be, somewhat paradoxically, associated with reduced trust in future research (Anvari and Lakens, 2018). This highlights a significant issue with the trustworthiness of research, which is exacerbated by a lack of enthusiasm.

In conclusion, as discussed in this contribution, the proposed remedies aimed to foster the credibility revolution are highly desirable and non-negotiable. However, these remedies (along with others yet to be envisaged) cannot, *sic et simpliciter*, be seen as a panacea for the replicability crisis without first being critically evaluated for their own hidden risks or costs. To avoid throwing the baby out with the bathwater, the scientific community desperately needs to find a new balance in the scientific process between imperative methodological rigor and best practices on the one hand and enthusiastic theory-advancing creativity and sustainability on the other. Both types of needs require more quality time for researchers, free from ill-conceived (and only apparently objective) institutional academic incentives and excessive bureaucratic burdens. We need to fine-tune best practices that ensure methodological thoroughness, without renouncing greasing the wheels of scientific creativity, while minimizing the overproduction of non-replicable results. Although I am certainly not the first to propose this sort of call to action, I hope that our journal, Frontiers in Psychology: Cognition, can serve (through specific Research Topics or other formats) as an inspiring receptacle for new ways of conceiving the entire scientific process in the pursuit of this new balance, free of any chauvinistic preconceptions.
